# Squamous cell carcinoma arising in a multiple verrucous epidermal nevus*

**DOI:** 10.1590/abd1806-4841.20164506

**Published:** 2016

**Authors:** Samira Yarak, Taila Yuri Siqueira Machado, Marilia Marufuji Ogawa, Mirian Luzia da Silva Almeida, Milvia Maria Simões e Silva Enokihara, Adriana Maria Porro

**Affiliations:** 1 Universidade Federal de São Paulo (UNIFESP) – São Paulo (SP), Brazil; 2 Private clinic – Luanda, Angola.

**Keywords:** Carcinoma, squamous cell, Nevus, Hamartoma, Skin neoplasms

## Abstract

Verrucous epidermal nevi are hamartomatous lesions of the epidermis that, unlike other epidermal nevi (such as sebaceous nevus or nevus comedonicus), are rarely associated with malignant neoplasms. The majority of squamous cell carcinoma develop in linear or multiple epidermal nevus and rarely in solitary epidermal nevus. In general, the prognosis is favorable. We report a case of well-differentiated invasive squamous cell carcinoma arising from a multiple verrucous epidermal nevus. Although there is no consensus on prophylactic removal of epidermal nevus, its removal and biopsy should be considered if changes occur.

## INTRODUCTION

Verrucous epidermal nevi (VEN) are hamartomatous lesions characterized by keratinocyte proliferation that arise from pluripotent cells in the germinal layer of the ectoderm. They are often present at birth or develop during childhood. In newborns they appear as velvety or brownish erythematous lines or plaques that evolve to hyperkeratosis and hyperpigmentation.^1^ The development of cancer is rare, with reported cases of keratoacanthoma, malignant eccrine poroma, basal cell carcinoma and squamous cell carcinoma (SCC).^2-5^ We report an unusual case of squamous cell carcinoma arising on multiple VEN.

## CASE REPORT

**Figure 1:**
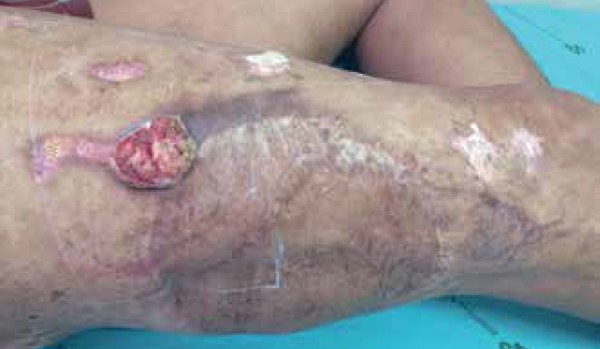
Ulcerated lesion on the right thigh with vegetative center at the anterior border of the graft.

**Figure 2:**
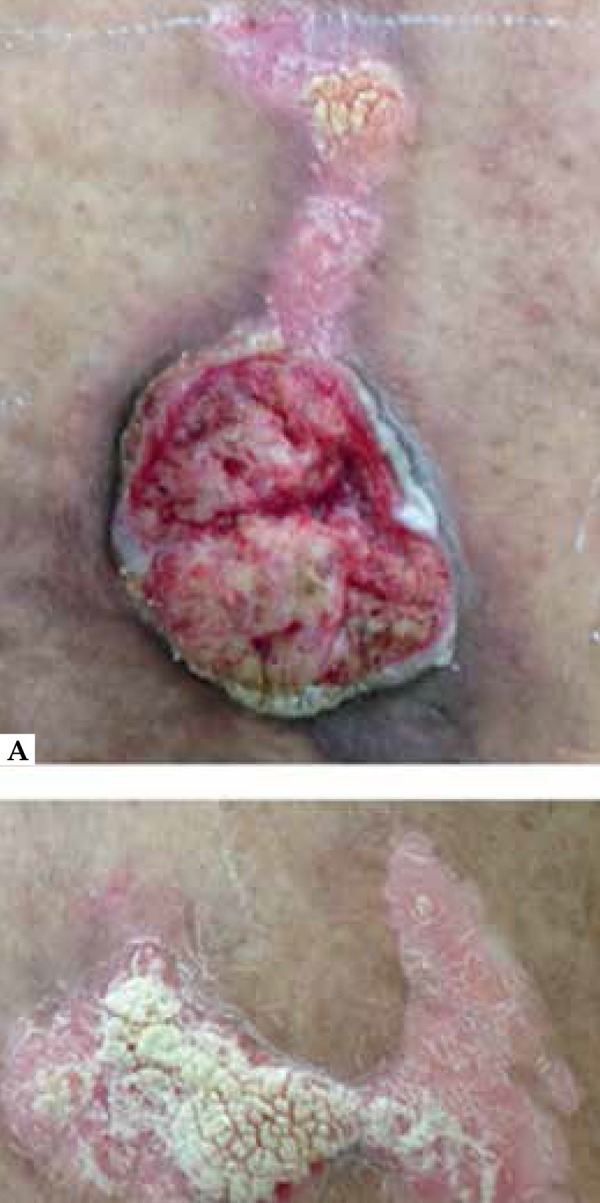
Right thigh lesions. A - ulcerated lesion in continuity with a erythematous keratotic plaque. B - Close up of Fig. A

**Figure 3:**
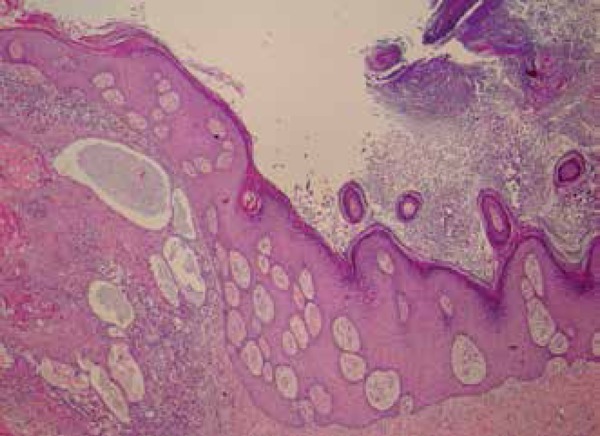
Excision of the tumor – on the left note the squamous cell carcinoma below the epidermal nevus area and the papillomatosis epidermal nevus (HE 40x) on the right.

A 37 year-old male presented un ulcerated 2-week-old 4.0 x 3.0 cm tumor near a surgical scar and erythematous keratotic plaques on the right thigh. Lymphadenopathy was not present (Figures 1 and 2). The patient had undergone surgical excision of skin cancer with partial graft (no histological exam) on the thigh four years before. Multiple erythematous keratotic lesions that had existed since childhood were reported. Skin biopsies of the lesions revealed well-differentiated invasive SCC associated with VEN spreading to the secretory portion of the eccrine glands. Nerve involvement, chronic inflammation and fibrosis were not reported. The polymerase chain reaction (PCR) for human papillomavirus (HPV) in the lesions was negative. Wide-local excision was performed and clear margins were obtained (Figure 3). No recurrence has been observed.

## DISCUSSION

VEN are a benign hyperplasia of the epidermis that, unlike other epidermal nevi (such as sebaceous nevus or nevus comedonicus), are rarely associated with malignant tumors. According to the literature, most SCC develop in linear or multiple epidermal nevus and rarely in solitary epidermal nevus.^6^ The main risk factors for the development of SCC are ultraviolet light, chemical carcinogens, chronic wounds, HPV infection and scarring of skin burns.^7^ SCC is the second most common type of nonmelanoma skin cancer. Although the claim that HPV causes SCC is still controversial, it might act as a carcinogenic co-factor, amplifying the risk of developing neoplasia.^8^ In our case, the presence of virus was negative. Several hypotheses have been proposed to explain the malignant transformation of chronic wounds. It is believed that persistent stimulation of chronic wounds can amplify growth and repair factors, which, in turn, may lead to malignancy.^9^ Perhaps local repetitive trauma can induce malignant transformation in VEN. The important prognostic factors for local recurrence or systemic metastasis for cutaneous SCC are tumor size, histological differentiation, tumor location and preceding injuries. For our patient, both the location of the tumor and differentiation were favorable for good prognosis. However, tumor size (4 cm) presented a higher risk of metastasis.

Riyadh *et al.* summarized 12 reported cases of SCC arising from VEN, revealing an equal male-to-female distribution. The mean age was 53.4 years and in half of the cases, the lesions were on the trunk; in the other half, on the limbs. In general, the prognosis was favorable based on lesion depth, degree of differentiation, perineural or perivascular infiltration factors that favor metastasis. Biopsy of the sentinel lymph nodes may be indicated.^10^

In conclusion, there is no consensus for prophylactic removal of VEN. However, the rate of malignancy in sebaceous nevus is low and the prognosis tends to be good, even in cases of malignant transformation. A similar strategy can be applied in the prophylactic removal of VEN and biopsy should be considered if changes occur in the VEN.
